# Evaluation of ionizing radiation as a risk factor for the incidence of breast cancer: long-term analysis after the cesium-137 accident in Goiânia, Brazil. An ecological study

**DOI:** 10.1590/1516-3180.2020.0041.R1.04052020

**Published:** 2020-08-14

**Authors:** Leonardo Bastos Lage, Ruffo Freitas-Junior, Rosangela da Silveira Corrêa, Eliane Eugênia dos Santos, Nilson Clementino Ferreira, Nivaldo Carlos Silva, Leonardo Ribeiro Soares

**Affiliations:** I MSc. Systems Analyst, Central-Western Regional Center for Nuclear Sciences, Comissão Nacional de Energia Nuclear (CNEN), Abadia de Goiás (GO), Brazil.; II MD, PhD. Professor, Mastology Program, Universidade Federal de Goiás (UFG), Goiânia (GO), Brazil.; III PhD. Senior Technologist, Central-Western Regional Center for Nuclear Sciences, Comissão Nacional de Energia Nuclear (CNEN), Abadia de Goiás (GO), Brazil.; IV PhD. Senior Researcher, Central-Western Regional Center for Nuclear Sciences, Comissão Nacional de Energia Nuclear (CNEN), Abadia de Goiás (GO), Brazil.; V PhD. Professor, School of Civil and Environmental Engineering, Universidade Federal de Goiás (UFG), Goiânia (GO), Brazil.; VI PhD. Physicist, Laboratório de Poços de Caldas (LAPOC), Comissão Nacional de Energia Nuclear (CNEN), Poços de Caldas (MG), Brazil.; VII MD, PhD. Physician, Mastology Program, Universidade Federal de Goiás (UFG), Goiânia (GO), Brazil.

**Keywords:** Radiologic health, Radiation, ionizing, Breast neoplasms, Incidence, Epidemiology, Brazil, Risk factors, Breast cancer, Goiânia, Goiás, Brazilian Breast Cancer Research Network, Goiânia (GO), Brazil

## Abstract

**BACKGROUND:**

The largest radiological accident to occur in any urban area happened in Goiânia, Brazil, in 1987.

**OBJECTIVE:**

To evaluate the association between breast cancer incidence and ionizing radiation levels.

**DESIGN AND SETTING:**

Ecological study among residents of the city of Goiânia, Brazil.

**METHODS:**

The central region of Goiânia, with seven major sources of contamination from cesium-137, was defined as the study area. The addresses of women diagnosed with breast cancer were identified between 2001 and 2010. The data were geographically referenced and, using census data, the annual averages of crude incidence rates were estimated. The existence of clusters of new cases was ascertained by means of the Moran index. Correlations of radiometric measurements with the incidence were assessed using unconditional linear regression.

**RESULTS:**

A total of 4,105 new cases were identified, of which 2,233 were in the study area, and of these, 1,286 (57.59%) were georeferenced. The gross rates of total and referenced cases were 102.91 and 71.86/100,000 women, respectively. These were close to the average for Brazilian state capitals, which is 79.37/100,000 women. The cluster analysis showed slight correlations in three small sets of census tracts, but these were far from the sources of contamination. The scatter plot of points and the R^2^ value close to zero indicated that there was no association between the variables.

**CONCLUSION:**

This study reinforces the hypothesis that the ionizing radiation levels to which women living in Goiânia are now exposed to are not associated with the onset of new cases of breast cancer.

## INTRODUCTION

Breast cancer is a public health problem in both developed and developing countries.^1^^,^^2^^,^^3^ According to the International Agency for Research on Cancer (IARC), there were two million new cancer cases and 626,000 deaths from breast cancer worldwide in 2018.^1^ In Brazil, breast cancer accounts for about 30% of all new cancer cases in women, annually.^2^ In this context, studies on risk factors are important for understanding the problem and for developing disease prevention policies.

Exposure to ionizing radiation is a well-known risk factor for the development of breast cancer. The risk is highest among young women at the time of exposure and is directly proportional to the radiation dose.^4^ In this context, there are several associations with genetic factors, which suggest that certain variations in deoxyribonucleic acid (DNA) damage-response genes can make it difficult for radiation-induced damage to be repaired.^5^ On the other hand, little is known about the impact of major radiological accidents on breast cancer incidence. The data from Japan and Chernobyl remain inconclusive, despite the increased risk of several other pathological conditions.^4^^,^^6^

In September 1987, a radiological accident of major proportions occurred in Goiânia, which affected both the population and the environment. The accident was caused by the removal and dismantling of a sealed source containing cesium-137, in an abandoned radiotherapy unit in Goiânia.^7^^,^^8^ The source was in the form of cesium chloride salt, which exhibited high solubility and was easily dispersible. The weather conditions at the time of the accident comprised heavy rain and a temperature of 26.4 °C. These conditions facilitated dispersion of the initial cesium-137 in the environment, around seven main spots in the central region of the city of Goiânia.^8^^,^^9^

It has been recognized that the greatest degree of contamination of the environment occurred in the areas of the main sources of contamination.^9^ It has therefore been speculated that the population neighboring these areas will have been subject to higher incidence of cancer than the general population. Hence, it is important to assess the temporal trends and possible existence of cancer clusters in these areas.^7^

Long-term and low-intensity environmental exposure is called chronic exposure. In such cases, knowledge of both the territorial distribution of emissions and the dynamics of the population transiting through the region enables calculation of the annual dose typically absorbed by an individual.^11^

## OBJECTIVE

Considering the cesium-137 accident and the increasing rates of breast cancer incidence in Goiânia, this study aimed to evaluate the spatial autocorrelation (clusters) of the addresses of women who had been diagnosed with breast cancer and ascertain the association between the incidence of breast cancer and the ionizing radiation levels to which the population was subjected.

## METHODS

An ecological study was conducted using the databases of new cases of breast cancer from the Population-Based Cancer Registry of Goiás (Registro de Câncer de Base Populacional de Goiás, RCBPGO) and radiometric measurements obtained with support from the National Nuclear Energy Commission (Comissão Nacional de Energia Nuclear, CNEN). To assess the existence of clusters and the association between the variables defined in this study, it was necessary to geographically locate the addresses of the women diagnosed with breast cancer. From this point, the crude incidence rates were calculated using the female population census data.

### Ethical issues

The study protocol was reviewed and approved by the internal review board of the Goiás Anticancer Association (number 31068514.43001.0031) and by the internal review board of the Universidade Federal de Goiás (UFG) (number 27742214.4.0000.5078). There was no need for informed consent.

### Study area

The city of Goiânia is the capital of the state of Goiás and, according to the 2010 census of the Brazilian Institute for Geography and Statistics (Instituto Brasileiro de Geografia e Estatistica, IBGE), the total population is 1,302,001. The territorial area of Goiânia is 733.116 km², with a population density of 1,776.74 inhabitants/km², and the city is divided into census tracts, which are the territorial units used for controlling collection of the census data.^12^

The local government also uses these territorial divisions in administrative and geographical units called healthcare districts, which are composed of a set of neighboring census tracts.

**Figure 1.**
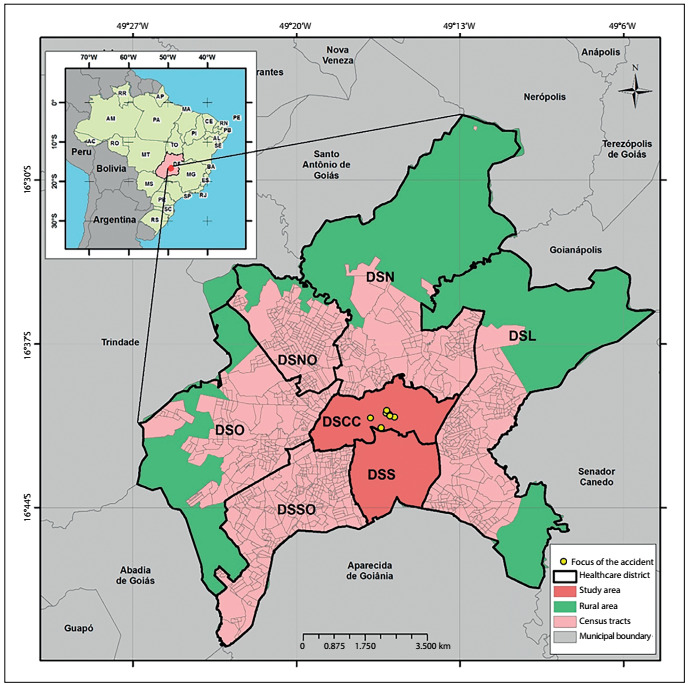
Division of the urban area of Goiânia into healthcare districts and respective census tracts, Goiânia, Goiás, Brazil, 2015.

The central region of Goiânia, comprised of the Campinas-Center and South healthcare districts, which contained the seven major sources of contamination of the cesium-137 accident and the majority of new cases of breast cancer, was defined as the study area, as shown in Figure 1.

Healthcare districts: DSN = North; DSL = East; DSNO = Northeast; DSCC = Campinas-Center; DSO = West; DSSO = Southwest; DSS: South.

### Data collection

#### Radiometric survey

The radiometric survey data and estimates of the doses to which the population of Goiânia was exposed were obtained from a previous study, conducted between 2010 and 2014.^13^ To conduct this survey, a mobile system for measuring gamma radioactivity in the environment, the Thermo Eberline FHT model 1376 MobiSys, was used. This system consisted of a high-sensitivity detector (five-liter plastic scintillator) coupled to a global positioning system (GPS) device and a microcomputer. The assembly was installed on a motor vehicle such that the height of the detector was one meter from the ground. The detector was configured to make absorbed dose rate measurements in the air every second. The data collected were the absorbed dose rate in the air, geographical coordinates, altitude and date and time of acquisition.^13^

#### Breast cancer incidence

From the RCBPGO database, information about new cases of breast cancer in the urban area of Goiânia that occurred between 2001 and 2010 was collected. The eligibility criteria for the records followed the RCBPGO methodology, comprising all cases of breast cancer diagnosed annually in women living in the city. To avoid inclusion of cases of women from other locations, the diagnosis of cancer needed to have been after the individual had settled in the city for a minimum period of six months. The consistency of the addresses in the records identified in the study area was checked and, whenever possible, they were confirmed from other public databases, with the aim of enabling their geographical referencing.

#### Crude incidence rate

To calculating the crude incidence rate of breast cancer, the number of new cases and the female population located in a specific geographical region were considered. Data from 2000 and 2010 demographic censuses were used, in accordance with the period covered by the survey. As defined by the IBGE, the census tract data comprised private households and people who were investigated as part of the entire population; these were, by convention, called universal results. The population data referred to the numbers of residents, in total and according to sex, in private and collective households.^12^

For comparison of the results between specific regions of Goiânia and even with other locations in Brazil, the indexes obtained from all cases were separated, considering the whole city, the study area and areas outside the study area. Moreover, for geographically referenced cases, the results were presented considering the study area and, in isolation, the two healthcare districts involved. From the number of new cases of breast cancer and the average number of women living in each specific geographical region, the annual crude breast cancer incidence rate (IR) per 100,000 women could be calculated, in accordance with equation (1).

$$IR\;=\;\left[(N\;/10)\;/\;P\right]\;\times \;100,000$$ (1)

IR -: Crude incidence rate of breast cancer.
N -: Number of new cases of breast cancer in the period from 2001 to 2010.
10 -: Number of years covered by the survey.
P -: Average female population, considering the 2000 and 2010 censuses.

To calculate the crude incidence rate, considering all the cases identified in the survey, the estimate of the average female population (P) was obtained by reducing the 2010 population by half the population change that was observed between 2000 and 2010 in the IBGE censuses, which was 19%; therefore, the reduction factor used was 9.5%. In addition, for the sample of geographically referenced cases, the average female population was also obtained by using a 9.5% reduction factor on the female population ascertained through the 2010 IBGE census and, in this case, only the census tracts in which there was at least one geographically referenced case were considered.

#### Cluster analysis

The complete addresses and their geographical coordinates that were identified as being within the study area were geographically referenced. We used a geographical information system (GIS), the ArcGIS 10.0 software, which was developed by the American company ESRI (Environmental Systems Research Institute) for spatial analyses and breast cancer incidence maps. A database was also generated per census tract in the study area, using the data on the number of women diagnosed with breast cancer.

Thus, it was possible to perform a spatial assessment of the incidence of new cases of breast cancer in a particular region within a defined period, and thus, to identify possible clusters. Such clusters are gathered from events that are not merely random and, from this, it is possible to draw comparisons between specific geographical areas.^14^

Using the database with the geographically referenced crude breast cancer incidence rates, the spatial autocorrelations in the census tracts within the study area were analyzed by means of the Moran index. This index is a correlation coefficient that measures the overall spatial autocorrelation, which is multidirectional and multidimensional. However, while other coefficients measure correlations ranging from perfect to no correlation, Moran’s is different: “-1” is a perfect clustering of dissimilar values (perfect dispersion); “0” is no autocorrelation (perfect randomness); and “+1” indicates perfect clustering of similar values (the opposite of dispersion).

#### Association between quantitative variables

The strength of the linear relationship between the continuous variables can be quantified using geographically weighted regression analyses. The main objective of this study was to analyze the association between the geographically referenced crude breast cancer incidence rates and the radiation dosimetry measurements according to census tract in the study area. We used linear regression analysis and calculation of R², which indicated the strength of the linear relationship between the variables using geographical weighted regression (GWR).

## RESULTS

### Breast cancer incidence

#### Total number of new cases

Between 2001 and 2010, 4,105 new cases of breast cancer were identified, among which 2,233 were in the study area and 1,872 were outside it. From the total number of new cases of breast cancer during the period of the survey, and the estimated average female population, the crude incidence rates were obtained and compared among specific regions and with estimates for 2016, as shown in Table 1.

**Table 1.** Comparison of the crude incidence rates per 100,000 women over the period from 2001 to 2010, in specific regions of Goiânia and other locations in Brazil

Geographical region	New cases of breast cancer	% of new cases in the sample	Average female population	Incidence rate per 100,000
Goiânia	4,105	100	616,435	66.59
Study area	2,233	54.40	216,981	102.91
Campinas-Center HD	1,053	25.65	108,108	97.40
South HD	1,180	28.75	108,873	108.38
Outside of study area^*^	1,872	45.60	399,454	46.86
Brazil^**^				56.20
State of Goiás^**^				52.09
Goiânia^**^				76.07

HD = healthcare district.

^*^In healthcare districts in Goiânia, other than those in the study area; ^**^Estimates for 2016.^3^

#### Number of geographically referenced new cases

In the study area, 2,233 new cases of breast cancer were identified in the period covered by the survey and, of these, 1,286 were geographically referenced, representing 57.59% of the total sample. The other cases did not present the necessary information for geographical referencing. The number of new cases of breast cancer ranged from one to 12 cases per census tract.

Considering only the sample of geographically referenced new cases and the average female population in each census tract (i.e. in tracts with at least one identified case), the crude incidence rates per 100,000 women were estimated. These are presented as aggregates in Table 2.

**Table 2.** Description of the crude incidence rates of new cases of breast cancer, geographically referenced per 100,000 women, according to sanitary district in the study area

Geographical region	New cases of breast cancer	Average female population (2000-2010)	Crude incidence rate per 100,000
Study area	1,286	178,950	71.86
Campinas-Center HD	569	84,009	67.73
South HD	717	94,941	75.52

HD = healthcare district.

The crude incidence rate in the sample of geographically referenced cases, in each census tract in the study area, ranged from 16.25 to 310.39 cases per 100,000 women in the Campinas-Center healthcare district and from 17.43 to 295.97 cases per 100,000 women in the South healthcare district.

#### Analysis of spatial clusters

**Figure 2.**
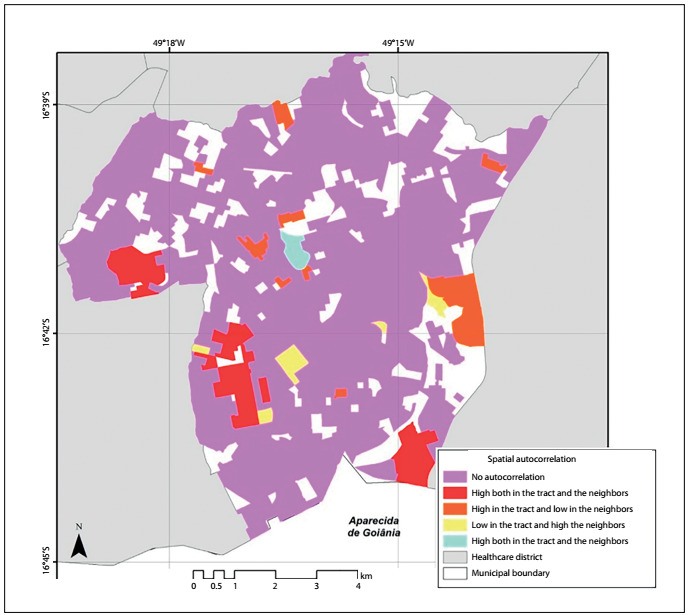
Spatial autocorrelation of the crude incidence rates of breast cancer (clusters) from 2001 to 2010, in each census tract of the Campinas-Center and South healthcare districts.

Through the statistical analysis on the spatial clusters considering the number of geographically referenced new cases of breast cancer, and from the crude incidence rates in each census tract, it was observed that most of the census tracts in the study area did not present any spatial autocorrelation. The crude incidence rates obtained in these tracts were statistically and geographically heterogeneous. There were three small sets of tracts, one in the southern region and two in the western region of the study area, where the cancer occurrence rate was high in several neighboring tracts, as seen in Figure 2.

#### Association between quantitative variables

From the geographically referenced databases of breast cancer crude incidence rates and radiometric measurements per census tract in the study area, it was possible to quantify the strength of the linear relationship between these variables.

#### Breast cancer incidence and ionizing radiation

**Figure 3.**
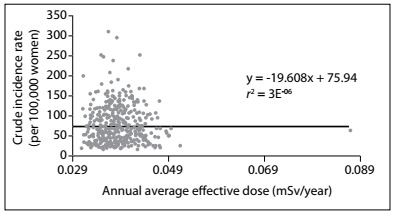
Association between the crude incidence rate of breast cancer and the annual average effective dose (mSv/year) in the census tracts of the study area (Campinas-Center South healthcare districts).

In order to evaluate the association between the variables studied, a linear regression analysis showing the dispersion of the points and R² values was performed, as displayed in Figure 3.

In the graph of Figure 3, the dispersion of the points does not follow any geometric pattern (line or curve) that would indicate any association between the variables. This can also be confirmed from the trend line, which is practically horizontal. The low R² values also indicate that there was a lack of association per census tract.

## DISCUSSION

All practices using ionizing radiation should observe the basic principles of radiation protection, including the principle of optimization. This concept dictates that all exposures should be kept as low as reasonably achievable (ALARA), since radiobiological and epidemiological studies on low doses have shown that there is no way to determine a dose threshold for the onset of stochastic biological effects.^15^

Evaluating a possible association between the incidence of breast cancer and the ionizing radiation levels existing in Goiânia is relevant, considering that both people and the environment in this area were seriously affected by the cesium-137 accident.^7^^,^^8^ This was the largest radiological accident that has occurred in any urban area and, therefore, it provides a rare opportunity to investigate the effects of ionizing radiation on occurrences of malignancies. In this context, this study reinforces the hypothesis that the radiological accident in Goiânia was not associated with the appearance of new cases of breast cancer.

The classical studies observed high cesium concentration at the incident site^16^ and indicated that exposure to ionizing radiation could be detected in descendants of the exposed individuals.^17^^,^^18^ Nevertheless, population-based studies with almost 30 years of follow-up have also suggested that was no association between the radiological accident and the incidence of cancer in the city.^19^^,^^20^ One possible explanation for this is that the genetic changes induced in the exposed population were not pathogenic or did not translate into increased cancer risk.^21^

In the overall analysis on breast cancer incidence in Goiânia, the peak incidence of the disease would have been expected in 1997, i.e. after a ten-year latency period following the accident. However, a recent study showed that the increase in the incidence of breast cancer in Goiânia was gradual between 1988 and 2004, with a tendency to stabilize after 2005.^20^ In this context, analysis on the incidence associated with the geospatial distribution pattern may identify points of higher concentration of the disease (clusters) and assist in constructing hypotheses about neoplasm behavioral change at that particular location.^22^^,^^23^^,^^24^

For the city of Goiânia, the estimates for 2016 showed that there were 250 new cases of breast cancer and a crude incidence rate of 76.07 cases per 100,000 women. Among the Brazilian state capitals, Goiânia presents an intermediate rate, close to the national average among the capitals, which is 79.37 new cases per 100,000 women.^2^ In addition, no abnormal pattern in the breast cancer incidence curve was observed in Goiânia over the past 30 years, compared with other Brazilian state capitals.^2^^,^^20^

Considering the total number of new cases of breast cancer identified in this study within the period between 2001 and 2010, the crude incidence rates per 100,000 women were much higher in the study area (102.91 cases per 100,000) than outside the study area (46.86 cases per 100,000), and higher than the average in Goiânia (66.59 cases per 100,000 women). Moreover, with regard to the absolute number of new cases, the study area presented a concentration of 54.40%, although the proportions in the Campinas-Center (25.65%) and South (28.75%) healthcare districts were similar.

Previous studies also identified higher concentrations of breast cancer cases in the Campinas-Center (39.95%) and South (20.48%) healthcare districts. These districts of Goiânia have populations with higher socioeconomic status, older women and easier access to healthcare services.^3^ Thus, the difference in the incidence rate may be due to greater adherence to screening programs and expression of other risk factors for the disease, such as age, obesity and hormone exposure.

Considering only the geographically referenced new cases of breast cancer in the study area and their respective healthcare districts, the crude incidence rates per 100,000 women were 71.86 in the study area overall, i.e. 67.73 in the Campinas-Center healthcare district, and 75.52 new cases per 100,000 women in the South healthcare district. Because the sample of geographically referenced cases represented 57.59% of the total identified, it was expected that the crude incidence rates obtained in the geographically referenced sample would be proportionately lower than the rates found for all the new cases identified in the survey, and this was confirmed.

For the cluster analysis, we considered the crude incidence rates that took into account both the number of new cases and the female population living in each census tract. Thus, one possible source of bias was eliminated, because if the absolute number of new cases of breast cancer were considered, a focus on more densely populated areas would be expected.

The data from this study add population-based information and geographical referencing involving breast cancer and exposure to cesium-137. In an individualized analysis on the direct victims of the accident, there was no difference in the overall incidence of cancer cases.^21^ By means of regression analysis, and considering the breast cancer crude incidence rate and the average dose of ionizing radiation, we found that there was no statistical association between these variables. However, new studies are needed in order to continue the evaluation of other risk factors associated with this neoplasm.

## CONCLUSIONS

This study reinforces the hypothesis that the ionizing radiation levels to which women living in Goiânia are exposed to are not associated with the onset of new cases of breast cancer. Discrete clusters were observed in specific regions away from the sources of contamination from the cesium-137 accident, which were the areas with the highest levels of ionizing radiation in Goiânia.
